# Mining the Impact of Mechanical-Stamping Heterogeneity on the Macro- and Micro-Levels of *Nongxiangxing daqu*

**DOI:** 10.3390/foods14213700

**Published:** 2025-10-29

**Authors:** Muwen He, Xiu Zhang, Ran Zhang, Bo Zhang, Rongqing Zhou, Chongde Wu, Chao Wang, Yi Dong, Yao Jin

**Affiliations:** 1 College of Biomass Science and Engineering, Sichuan University, Chengdu 610065, China; helpmoonwood@163.com (M.H.); orangeran_7@163.com (R.Z.); zhangbo20182022@163.com (B.Z.); zhourqing@scu.edu.cn (R.Z.); cdwu@scu.edu.cn (C.W.); 2 Key Laboratory of Leather Chemistry and Engineering, Ministry of Education, Sichuan University, Chengdu 610065, China; 3 Luzhou Lao Jiao Co., Ltd., Luzhou 646699, China; zhangxiu1@lzlj.com (X.Z.); wangchao5@lzlj.com (C.W.); 4 National Engineering Research Center of Solid-State Brewing, Luzhou 646699, China

**Keywords:** *nongxiangxing daqu*, *daqu* production craft, mechanical stamping, physicochemical properties, microbial community

## Abstract

In the production of modern *nongxiangxing daqu*, mechanical stamping is utilized to compact raw materials into *daqu* bricks. Nevertheless, variations in stamping frequencies may modify the initial physicochemical properties of *daqu*, which in turn influence its physicochemical and biochemical parameters, and ultimately affect the quality of *baijiu*. This study systematically evaluated *daqu* samples prepared with different stamping frequencies (2 to 5 cycles) in terms of (1) physicochemical and biochemical parameters, (2) volatile compound profiles, (3) microbial community dynamics, and (4) interspecific interactions. The results showed that with the increase in stamping frequency, the moisture content, fermentative power, esterifying power, and liquefying power of daqu were all enhanced, with respective increases of 20.11%, 67.16%, 12.24-fold, and 36.27%. Specifically, the relative abundances of *Weissella*, *Lactobacillus*, *Aspergillus*, and *Rasamsonia* in *daqu* exhibited a significant increase with the elevation of pressing cycles. With the reduction in stamping frequency, the primary producers of flavor compounds shifted gradually from bacteria to fungi. These findings verify that stamping frequency exert a substantial regulatory impact on the physicochemical and biochemical parameters, microbial community dynamics, accumulation of flavor substances, and abundance of functional enzymes in *daqu*. Through a systematic elucidation of the mechanistic links between stamping parameters and *daqu* functionalities, this research offers actionable insights for optimizing industrial pressing processes and establishes a scientific basis for modern *daqu* production.

## 1. Introduction

In the realm of *nongxiangxing baijiu* production, *nongxiangxing daqu* serves as an indispensable starter, playing a crucial role in multiple facets of the brewing process [1]. It provides essential raw materials, facilitates the decomposition of polysaccharides, catalyzes the fermentation of ethanol, and contributes significantly to the overall flavor profile of the *baijiu* [2]. The production sequence of *nongxiangxing daqu* can be systematically divided into four primary stages: the initial crushing and wetting of raw materials, followed by compression molding, temperature-controlled fermentation, and ultimately, room temperature storage [3,4].

Within this production framework, the methods employed for shaping *daqu* can be categorized into two distinct approaches: traditional manual trampling and contemporary mechanical stamping [5,6,7]. Historically, the production of *nongxiangxing daqu* was predominantly reliant on manual press techniques, where the quality of the final *daqu* products was largely contingent upon human experience and judgment [8]. This led to notable inconsistencies in the brewing effects observed among different batches of *daqu* [6]. Nevertheless, with the steady advancement of technology, the production paradigm of *nongxiangxing daqu* has undergone a significant transformation, gradually shifting from manual production methods to mechanized processes [9,10]. Prior research endeavors have demonstrated that mechanical production of *daqu* not only enhances operational efficiency when compared to manual methods but also ensures a greater degree of consistency in terms of *daqu* quality, sensory attributes, and brewing outcomes [11,12,13]. This shift towards mechanization has been pivotal in addressing the inconsistencies observed in manually produced batches, thereby enhancing the overall predictability and reliability of the *baijiu* brewing process.

Currently, due to the rapid advancements in mechanization and artificial intelligence technologies, mechanical intelligence technology is highly favored by Chinese *baijiu* enterprises [4,14,15]. In the production, handling, and storage of *daqu*, fully unmanned operations have been achieved. This trend has led to the gradual replacement of traditional handmade *daqu* by mechanically produced *daqu*. However, whether mechanically produced *daqu* can fully replace handmade *daqu* has become a major issue [16,17]. Therefore, exploring the differences between mechanically produced *daqu* and handmade *daqu* has become a research hotspot among *baijiu* researchers. Huang et al. [5] conducted a detailed analysis of the physicochemical indicators of mechanically produced *daqu* and handmade *daqu*, discovering that the physicochemical indicators of mechanically produced *daqu* are more stable. Furthermore, Mu et al. [7] performed a comparative analysis of Jiangxiangxing *daqu* produced via traditional and mechanical methods, revealing that the mechanically produced *daqu* excelled in acidity, amino acid nitrogen content, enzyme activity, and volatile substances, outperforming the handmade *daqu*. The aforementioned studies agree that the mechanized production of *daqu* is more conducive to microbial succession and the generation of metabolic components. Hence, mechanical production has become the predominant production methodology for *daqu*.

At present, there are three main categories of apparatuses for *daqu* production available in the market, namely hydraulic, pneumatic, and mechanical stamping. When compared to hydraulic and pneumatic stamping *daqu* apparatuses, mechanical stamping apparatuses have the merits of synchronous providing raw material and *daqu* manufacturing, multiple stamping and shaping, and evading the hazard of contaminating *daqu* due to oil leakage. The stamping *daqu* machine is outfitted with several rectangular molds that possess regular geometries. Once the first mold accomplishes the preliminary shaping, the raw material for *daqu* is sequentially pressed by the remaining molds under the conveyor of the track, thereby obtaining the final formed *daqu* blocks. Mechanical *daqu*, after undergoing stable molding and multiple stamping procedures, exhibits a unified and stable microbial community and physicochemical indicators [13,18]. Nevertheless, the existing research regarding mechanical *daqu* predominantly centers on fixed stamping frequency, there has been a dearth of studies on the differences in mechanical *daqu* with various stamping frequencies. Thus, whether modifying the stamping frequency impacts the quality of the final *daqu* product remains unclear and is an area that warrants further academic exploration.

Therefore, to systematically investigate the impact of stamping frequency on *daqu* properties, we employed a *daqu*-stamping machine with adjustable stamping plates, four different *daqu* (2, 3, 4, and 5 stamping frequency) were obtained by reducing the number of stamping plates (Figure S1). The resulting *daqu* blocks underwent standardized fermentation and maturation for three months under controlled environmental conditions. Through longitudinal monitoring and comprehensive sampling across the entire fermentation period, we conducted multiscale analyses (macro- and microscopic) to elucidate the differential effects of varying stamping frequencies on *daqu* characteristics, including sensory attributes and functional capabilities.

This study provides a comprehensive understanding of how stamping heterogeneity governs quality development in *nongxiangxing daqu*. By elucidating the fundamental relationships between mechanical processing parameters and *daqu*’s biochemical characteristics, our findings establish a scientific framework for precision control of industrial stamping operations and intelligent manufacturing system optimization. These advances not only deepen the mechanistic knowledge of traditional fermentation ecology but also bridge critical gaps between empirical craftsmanship and modern automated production technologies.

## 2. Materials and Methods

### 2.1. Daqu Stamping and Samples Collection

In order to obtain *daqu* with different stamping frequencies, *daqu* was produced in an enterprise in Luzhou City, Sichuan, China (28°92′ N, 105°48′ E). The stamping frequency was systematically controlled by adjusting the number of stamp plates installed on the *daqu* stamping machine, generating four experimental groups with 5, 4, 3, and 2 stamping cycles. The resulting *daqu* blocks were correspondingly labeled as YZ-5, YZ-4, YZ-3, and YZ-2 based on their respective stamping frequency (Figure S1). The stamped *daqu* was placed in the fermentation room for 14 days, and then transferred to the storage room for 90 days to obtain mature *daqu*.

Three parallel bricks were taken out from the same room on days 0, 4, 8, 14, 22, 30, 60, and 90, respectively. They were labeled YZ-5 0d, YZ-4 0d, YZ-3 0d, etc., according to stamping frequency and sampling time. These *daqu* samples were individually mashed to form powders, then placed into sterile bags. Subsequently, they were stored at −80 °C until further analyses were carried out.

### 2.2. Physiochemical Properties Determination

The physiochemical characteristics of *daqu* samples, such as water content, acidity, liquefying power, saccharifying power, fermentative power, and esterifying power were ascertained in accordance with the previous general methods [19]. Each of the *daqu* samples was subjected to measurement on three separate occasions to ensure accuracy and reliability of the obtained data.

### 2.3. Determination of Volatile Compounds in Daqu by HS-SPME-GC-MS

The volatile components present in *daqu* were determined through the utilization of headspace solid phase microextraction gas chromatography-mass spectrometry (HS-SPME-GC-MS). For the extraction of volatile compounds, a three-phase extraction head with a 50/30 μm DVB/CAR/PDMS fiber (manufactured by Supelco, Inc., Bellefonte, PA, USA; purchased from Sigma, Beijing, China) was employed. In brief, 1.0 g *daqu* powder was placed into a 15 mL headspace bottle, and subsequently, 10 μL of an internal standard (2-octanol, with a concentration of 0.0474 g/100 mL) was added. The headspace bottle was then positioned in a constant temperature stirrer, preheated, and allowed to reach equilibrium at 60 °C for a duration of 15 min. Following this, the SPME fiber was exposed to the headspace within the bottle at 60 °C for 50 min.

After the sampling process was completed, the fiber was introduced into the injector of the gas chromatograph and left there for 3 min to enable the thermal desorption of the analytes. The volatile compounds were then separated using a Shimadzu (Japan) GCMS-QP2010SE system. The temperature-rise procedure for the oven was set as follows: it was maintained at 40 °C for 5 min, then increased to 220 °C at a rate of 5 °C/min, and finally held at 220 °C for an additional 5 min.

The mass spectrometer was operated in the electron ionization mode with an energy of 70 ev, and the mass spectral scanning range was configured to be from 40 to 500 amu. The constituents of the volatile components were identified by comparing their mass spectra with those in the NIST05 spectrum database.

### 2.4. Extraction, PCR Amplification, and Illumina MiSeq Sequencing of Microbial DNA from Daqu

Extract microbial community genomic DNA from *daqu* samples using a DNA kit (Omega Bio-tek, Norcross, GA, USA). DNA extract was examined in 1% agarose gel, and DNA concentration and purity were determined with NanoDrop 2000 UV vis spectrophotometer (Thermo Fisher Scientific, Waltham, MA, USA). The highly variable region V3–V4 of the 16S rRNA gene in bacteria was amplified using a ABI GeneAmp^®^ 9700 PCR thermal cycler (manufactured by Thermo Fisher Scientific, Waltham, MA, USA) using primers 338F (5′-ACTCCTACGGGGCAG-3′) and 806R (5′-GGACTACHVGGGTWTCTAAT-3′), while the fungal ITS region was amplified using primers ITS1F (5′-CTTGGTCATTTAGAGGAGAGTAA-3′) and ITS2R (5′-GCTGCGTTCTTCATCGATGC-3′). The PCR amplification process is as follows: initial denaturation at 95 °C for 3 min, followed by denaturation at 95 °C for 30 s, annealing at 55 °C for 30 s, extension at 72 °C for 45 s, single extension at 72 °C for 10 min, and end at 4 °C. PCR mixture contains 5 × TransStart FastPfu Buffer 4 μL. 2.5 mM dNTPs 2 μL. Forward primer (5 μM) 0.8 μL. Reverse primer (5 µM) 0.8 µL, DNA polymerase 0.4 μL. Template DNA 10 ng, ddH2O 20 μL. PCR products were extracted from 2% agarose gel, purified with DNA gel extraction kit (Axygen Biosciences, Union City, CA, USA) according to the manufacturer’s instructions, and quantified with Quantus fluorometer. Send the extracted DNA to Major Biotechnology Co., Ltd. (Shanghai, China) for paired sequencing on the Illumina MiSeq PE300 platform/NovaSeq PE250 platform.

### 2.5. Sequence Analysis

The QIIME (v1.8.0) pipeline was utilized to handle sequencing data [20]. The low-quality sequences were filtered out based on the following criteria, sequences with a length of less than 150 bp; sequences having an average Phred score of less than 20; sequences that contained ambiguous bases; and sequences that had mononucleotide repeats of more than 8 bp [21,22]. The paired-end reads were assembled by means of FLASH (v 1.2.11) [23]. After the detection of chimeras, the remaining high-quality sequences were grouped into operational taxonomic units (OTUs) with a 97% sequence similarity level using UCLUST (v 1.1.579) [24]. A representative sequence was chosen from each OTU with the default parameters. The taxonomic classification of OTUs was carried out by conducting BLAST searches of the representative sequence set against the NCBI NT Database, taking the best hit [25]. An OTU table was further created to record the abundance of each OTU in each sample as well as the taxonomy of these OTUs. OTUs that accounted for less than 0.001% of the total sequences across all samples were discarded. To reduce the disparity in sequencing depth among samples to the minimum, an averaged, rounded OTU table was generated. This was achieved by averaging 100 evenly resampled OTU subsets under 90% of the minimum sequencing depth, which was then used for further analysis.

### 2.6. Bioinformatics and Statistical Analysis

Co-occurrence analysis was performed by calculating Spearman’s rank correlations between predominant taxa. Correlations with |RHO| > 0.6 and *p* < 0.01 were visualized as co-occurrence network using Cytoscape (v 3.10.1). Taxa abundances at the genus levels were statistically compared among samples or groups by Metastats and visualized as columnar stacking diagram. Statistical analyses were performed using SPSS software v. 27.0.1 (SPSS Inc., Chicago, IL, USA).

## 3. Results and Discussion

### 3.1. Effect of Stamping Frequency on the Physicochemical Properties of Daqu

**Figure 1:**
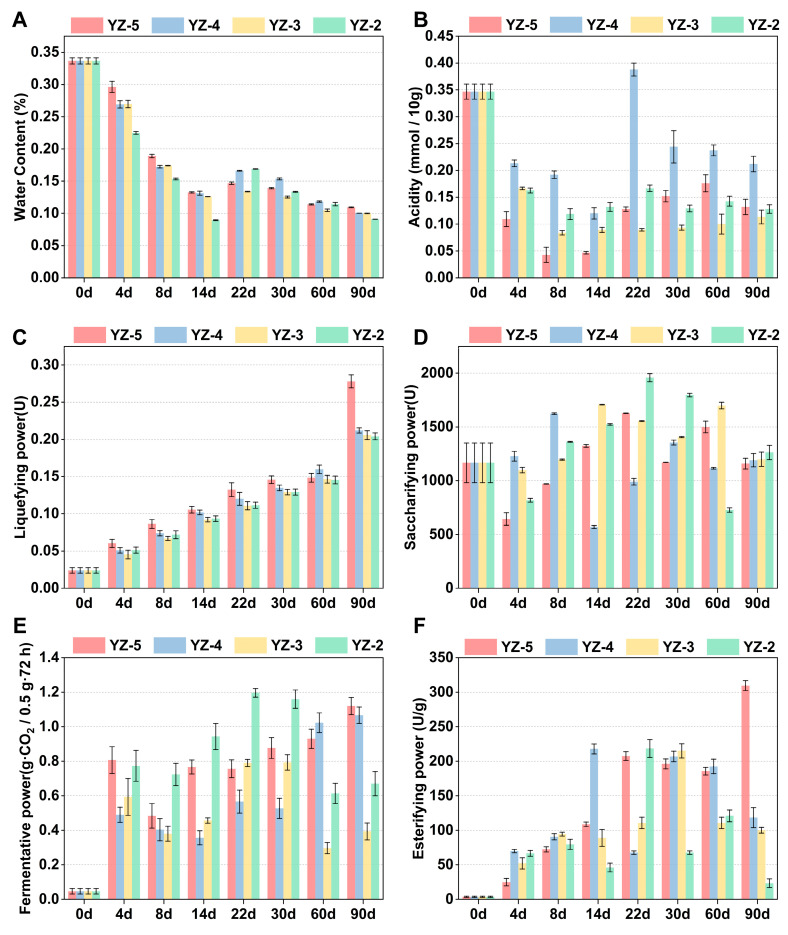
Changes in physicochemical indicators of *daqu* with different stamping frequencies. (**A**) Water content. (**B**) Acidity. (**C**) Liquefying power. (**D**) Saccharifying power. (**E**) Fermentative power. (**F**) Esterifying power.

To elucidate the impact of varying stamping frequencies on these indices, a comprehensive analysis was conducted encompassing water content, acidity, liquefying power, saccharifying power, fermentative power, and esterifying power across distinct *daqu* samples. The results revealed a decline in water content with prolonged fermentation time (Figure 1A), aligning with prior research findings reported in the literature [26]. Specifically, an increase in stamping frequency is associated with enhanced water retention capacity of *daqu*, a phenomenon that is particularly evident during the fermentation stage. This phenomenon can be attributed to the gradual rise in environmental temperature during fermentation: a higher stamping frequency reduces *daqu* porosity, strengthens its water retention capacity, and thereby slows down water evaporation [9]. Additionally, reduced stamping frequency decreased *daqu* density (Figure S2), potentially explaining the decline in its water content. However, *daqu* density had no significant effect on its acidity. The acidity showed an overall decreasing trend within 0–14 days and began to rise after entering the storage period (day 22) (Figure 1B). Additionally, *daqu* with more stamping frequencies demonstrates superior liquefying, fermentative, and esterifying power, but stamping frequency did not result in a significant change in saccharifying power of all four types of *daqu* remained at approximately 1000 U (Figure 1C–F). The discrepancy in multi-capabilities in *daqu* regarded as an indicator of the different metabolic activities extent undertaken by microorganisms [27].

Prior studies have shown that the disparities between artificial *daqu* and mechanical *daqu* result from the inhomogeneity of density, porosity and uniformity [28]. Reducing the times of mechanical stamping, while guaranteeing uniform compression, enlarges the porosity of *daqu*, making the interior more porous, approaching the “tight outside and loose inside” feature of artificial *daqu*.

The increase in the porosity of *daqu* accelerates the gas exchange rate between the interior and the exterior of *daqu*, which hastens the loss of moisture in *daqu*. Meanwhile, the loose and porous structure is conducive to the growth of filamentous fungal spores. Filamentous fungi have been repeatedly verified in previous studies as the main microorganisms responsible for glucoamylase production and are closely related to the saccharifying power of *daqu* [21,29]. Prior investigations had demonstrated that the saccharifying power of artificial *daqu* was substantially higher than that of mechanical *daqu*, this is also attributed to the differences in porosity. In previous studies, esterifying power, liquefying power and fermentative power were confirmed to be related to yeasts including *Pichia*, *Saccharomycopsis*, and Wickerhamomyces in *daqu* [30,31]. The richness of yeasts in mechanical *daqu* is considerably higher than that in artificial *daqu*.

### 3.2. Effect of Stamping Frequency on the Volatile Compounds in Daqu

**Figure 2:**
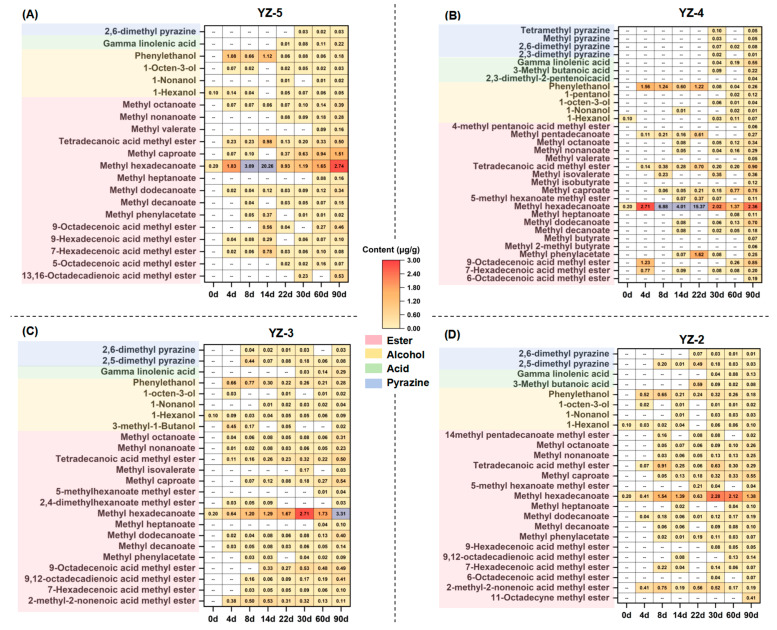
Volatile composition and content of *daqu* under different stamping configurations: (**A**) Stamping frequency = 5; (**B**) Stamping frequency = 4; (**C**) Stamping frequency = 3; (**D**) Stamping frequency = 2.

Figure 2 illustrates the composition of volatile compounds in distinct stamping frequencies *daqu*. Specifically, total 56 esters, 13 alcohols, 6 acids and 5 pyrazines were detected in different *daqu* (Table S1). In the mature *daqu* of YZ-5, YZ-4, YZ-3 and YZ-2 (after 90 days), ester compounds are the predominant volatile compounds, the quantities of ester compounds were 15, 20, 16, and 17, respectively (Figure 2), with ester concentrations reaching 7.48 μg/g, 8.21 μg/g, 6.84 μg/g, and 4.17 μg/g correspondingly. Figure 1B,E indicate that YZ-4 has the highest acidity and YZ-5 has the highest esterifying power, which might be among the reasons why YZ-5 and YZ-4 have relatively higher ester concentrations.

Among ester compounds, methyl hexadecanoate is the absolutely dominant compound, accounting for over 80% of the concentration in all four groups of *daqu*, which is in line with previous research findings. However, significant differences exist in the time and concentration when methyl hexadecanoate peaks in *daqu* with various stamping frequencies. YZ-5 reached its peak concentration (20.26 μg/g) at 14 d of fermentation, while YZ-4 peaked (15.37 μg/g) at 22 d. YZ-3 and YZ-2 achieved their maximum values (3.31 μg/g and 2.28 μg/g) at 90 d and 30 d, respectively. The reduction in stamping frequency not only decreases the abundance of dominant volatile compounds but also postpones the time at which they reach their peaks. This might be due to the fact that a decreased stamping frequency increases *daqu* porosity, leading to the esters in *daqu* being difficult to retain and prone to volatilization. It has been proven by prior research that artificial *daqu*, which has a relatively high porosity, has a lower concentration of volatile compounds than mechanical *daqu* with low porosity. Simultaneously, mechanization can promote the synthesis of volatile substances in *daqu* [7]. The higher stamping frequencies, the more conducive it is to the synthesis of ester compounds. The content changes in other volatile compounds are not significantly different, indicating that the alteration of stamping frequency does not markedly change the concentrations of volatile compounds with low concentrations. In conclusion, reducing the stamping frequency will lead to the loss of dominant volatile compounds and decrease the flavor quality of *daqu*.

### 3.3. Effect of Stamping Frequency on Microbial Community in Daqu

**Figure 3:**
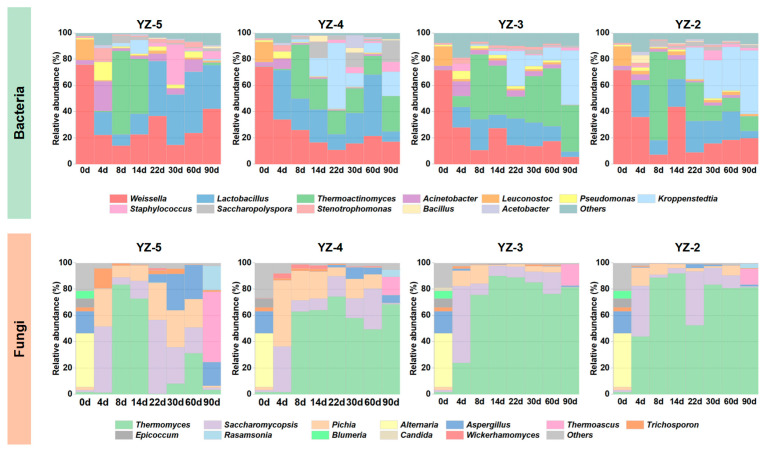
Microbial community composition and succession of *daqu* with different stamping configurations.

Figure 3 exhibits the composition and succession of *daqu* bacterial and fungal communities at various stamping frequencies. In the bacterial community, *Weissella*, *Lactobacillus*, *Thermoactinomyces*, and *Kroppenstedtia* are the predominant bacterial genera in *daqu*, which is in line with prior studies [29,32]. Nevertheless, the different stamping frequencies lead to variable bacterial compositions and successions in different *daqu* groups. Upon entering the storage period (22–90 d), *Weissella* and *Lactobacillus* are the dominant bacteria in YZ-5 *daqu*, while YZ-4, YZ-3, and YZ-2 also have *Kroppenstedtia* as the dominant microorganism. At the conclusion of storage (90 days), the relative abundance of *Weissella* and *Lactobacillus* in YZ-5 was considerably higher than that in the other three groups of *daqu*, whereas the abundances of *Kroppenstedtia* and *Thermoactinomyces* were much lower than in the other three groups of *daqu*.

Regarding fungi, consistent with previous investigations, *Thermomyces*, *Saccharomycopsis*, and *Pichia* are the dominant fungi in *daqu* [33,34]. According to Figure 3 the fungal community composition of *daqu* was approximately identical during the fermentation period (0–14 d). Yeast fungi (*Saccharomycopsis*, *Pichia*) abruptly increased in the early fermentation stage (4 d) and gradually began to be replaced by *Thermomyces* as the fermentation room temperature rose [2]. However, after entering the storage period (22 d), *Thermomyces* remained the principal dominant fungi in YZ-4, YZ-3, and YZ-2, while *Thermomyces* in YZ-5 were nearly nonexistent and were replaced by *Saccharomycopsis*, *Pichia*, and *Aspergillus* as dominant microorganisms. At the end of storage (90 days), the fungal community structure of YZ-5 demonstrated significant differences compared to YZ-4, YZ-3, and YZ-2. *Thermoascus*, *Rasamsonia*, and *Aspergillus* in YZ-5 were significantly higher than in the other three groups, while *Thermomyces* were significantly lower than in the other three groups.

Based on the community successions of bacteria and fungi, it was discovered that the diverse stamping frequencies not only modified the dynamic successions of microorganisms but also altered the compositions of microbial communities in mature *daqu*. *Lactobacillus* and *Weissella*, as the two primary anaerobic lactic acid bacteria in *nongxiangxing daqu*, possess the ability to convert carbohydrates into lactic acid and secrete antibacterial compounds to inhibit the growth of most bacteria [35,36]. *Thermoactinomyces* and *Kroppenstedtia* have been identified as thermophilic aerobic bacteria in previous studies [37,38]. LAB and thermophilic bacteria have different requirements for temperature and oxygen, resulting in a diverse succession pattern of growth and decline.

Similarly, in the case of fungi, *Thermomyces*, as a thermophilic fungus, has a similar trend of change to *Thermoactinomyces* and *Kroppenstedtia*. Conversely, *Saccharomycopsis* and *Pichia* have lower heat tolerance [39], so their trend of change is opposite to that of heat-resistant fungi. From Figure 3, it can be observed that a high stamping frequency is not conducive to the growth of heat-resistant microorganisms. This might be because the more stamping frequencies there are, the smaller the porosity of *daqu*, leading to a slower heat exchange rate. The temperature inside *daqu* is beneficial for maintaining stability, which is advantageous for the growth of LAB and filamentous fungi but not suitable for the growth of thermophilic microorganisms. Previous studies have indicated that mechanical *daqu* and artificial *daqu* differ in microbial succession due to differences in porosity [5,7].

Figure S3 shows the changes in microbial community diversity of *daqu* with different stamping frequencies. The Ace, Chao1, and Shannon indices reflect the richness (species number) of microorganisms in Daqu. The fungal richness of 0 day *daqu* was higher than that of bacteria; as fermentation proceeded, bacteria gradually replaced fungi, and the bacterial richness in mature *daqu* was higher than that of fungi, indicating that the total number of bacteria increased while the number of fungi decreased during fermentation. Meanwhile, the reduction in stamping frequency inhibited the microbial richness in *daqu*. The Chao1 index, Ace index, and Shannon index of mature YZ-5 *daqu* were higher than those of other *daqu* samples, and this characteristic was more prominent in terms of fungal diversity, suggesting that the reduction in stamping frequency leads to a decrease in microbial quantity. In addition, the Simpson index represents the microbial diversity in *daqu*. During fermentation, the fungal diversity in *daqu* gradually increased while the bacterial diversity gradually decreased; in mature *daqu*, the bacterial/fungal diversity of YZ-5 *daqu* was lower than that of other *daqu* samples. These results indicate that during the fermentation of *daqu*, dominant bacterial strains emerged in the bacterial community, inhibiting the growth of other bacteria and resulting in a reduction in bacterial diversity. In contrast, the dominant position of fungi was gradually diminished, and various fungi began to co-grow, ultimately leading to high fungal diversity in *daqu*.

### 3.4. Microbe-Centric Correlations Across Stamping Frequencies

#### 3.4.1. Microbial Interaction Network

**Figure 4:**
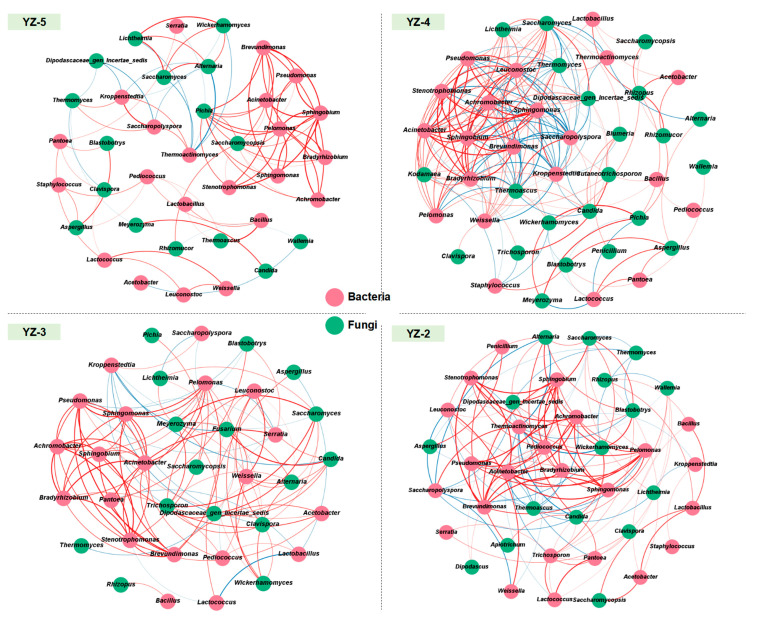
The microbial interaction relationship of *daqu* with different stamping configurations. A connection stands for a statistically significant (*p* < 0.05) and strong positive (red, Spearman’s Rho > 0.7) or negative (blue, Spearman’s Rho > −0.7) correlation. The nodes are colored by group, and thickness of edges is proportional to the absolute value of Spearman’s correlation coefficients.

Figure 4 shows the microbial interaction relationships of *daqu* with different stamping frequencies. Different stamping frequencies result in different degrees of complexity of interaction relationships in *daqu*. Generally, there are 101, 203, 104, and 133 pairs of interaction relationships in YZ-5, YZ-4, YZ-3, and YZ-2 *daqu*, respectively. However, there are relatively few interaction relationships between the dominant bacteria *Weissella*, *Lactobacillus*, *Thermoactinomyces*, *Kroppenstedtia* and the dominant fungi *Thermomyces*, *Saccharomycopsis*, *Pichia*, *Aspergillus*. Among the 8 dominant microorganisms, there are only 47, 70, 21, and 33 pairs of interaction relationships in YZ-5, YZ-4, YZ-3, and YZ-2, respectively.

In general, the interaction relationships among the dominant microorganisms in different *daqu* are similar. Thermophilic microorganisms show a negative correlation with other non-thermophilic microorganisms [40,41], and yeasts show a positive correlation with yeasts and lactic acid bacteria [42]. The change in stamping frequency will affect the interaction relationships in the microbial community as a whole, but it cannot significantly change the interaction relationships between dominant microorganisms. The interaction relationships between non-dominant microorganisms and dominant microorganisms are significantly affected by the stamping frequency.

#### 3.4.2. Correlation Between Microorganisms and Physicochemical Indexes

**Figure 5:**
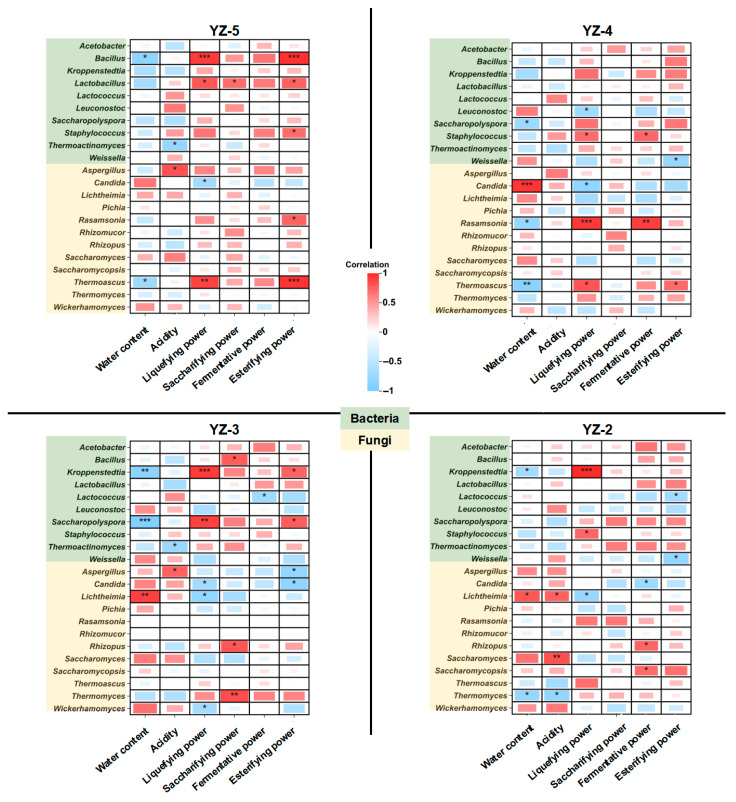
The interaction between internal microorganisms and physicochemical indicators of *daqu* with different stamping configurations (* *p* < 0.5, ** *p* < 0.05, *** *p* < 0.01).

Figure 5 illustrates the correlations between the 23 major microbial genera in *daqu* and its physicochemical and biochemical indices. Variations in stamping frequencies significantly altered the correlations between microorganisms and physicochemical/biochemical indices in *daqu*. In general, compared with other *daqu*, YZ-5 exhibited more positive correlations between microorganisms and fermentative power, saccharifying power, and esterifying activity. Specifically, in YZ-5, *Aspergillus* showed positive correlations with acidity, fermentative power, saccharifying power, and esterifying power. However, the positive correlations of *Aspergillus* gradually weakened with the decrease in pressing cycles. In YZ-2, *Aspergillus* even displayed negative correlations with fermentative capacity and esterifying activity. Similarly, in YZ-5, *Lactobacillus* and *Bacillus* presented strong positive correlations with fermentative power, saccharifying power, and esterifying power, whereas such correlations were weaker in other *daqu* samples. In YZ-5, the correlations between *Rhizopus*, *Saccharomycopsis*, and biochemical indices were relatively weak; in contrast, these correlations were enhanced in YZ-2.

According to the above results, it can be seen that the change in stamping frequency will change the dominant position of microorganisms in *daqu*. In YZ-5, physicochemical and biochemical indices are mainly dominated by *Lactobacillus*, *Bacillus* and *Aspergillus*. While in YZ-2, dominant *Rhizopus* and dominant *Saccharomycopsis* become the microorganisms dominating physicochemical and biochemical indices. This may be related to the internal structure of *daqu*. The more stamping frequencies, the closer the interior of *daqu* is to an anaerobic environment, which is conducive to the growth of anaerobic microorganisms *Lactobacillus* and *Bacillus* [43]. *Rhizopus* belongs to aerobic fungi [44], the fewer stamping frequencies, the more oxygen there is inside *daqu*, which is more conducive to *Rhizopus*. *Saccharomycopsis* belongs to facultative anaerobe [45], different abilities will be exhibited under different oxygen contents. In the YZ-5, *Saccharomycopsis* may carry out more anaerobic respiration, enhancing the fermentative power of *daqu*, which explains the reason for the high fermentative power of YZ-5 in Figure 1E.

#### 3.4.3. Correlations Between Microorganisms and Volatile Compounds

**Figure 6:**
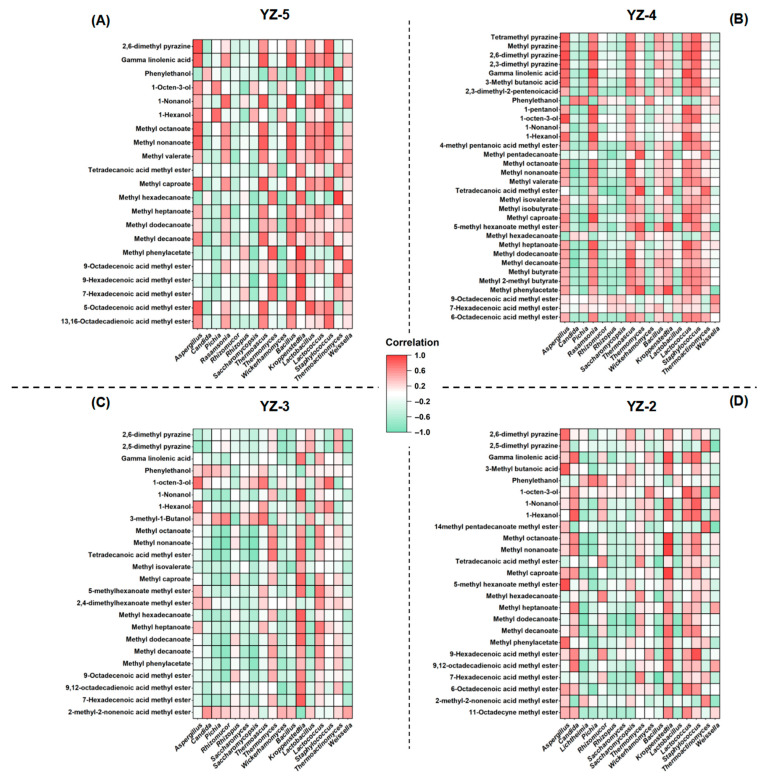
Correlation of microbial communities and volatile compounds in different stamping frequencies *daqu*: (**A**) Stamping frequency = 5; (**B**) Stamping frequency = 4; (**C**) Stamping frequency = 3; (**D**) Stamping frequency = 2.

The interaction relationships between the microorganisms and volatile compounds in *daqu* with different stamping frequencies are shown in Figure 6. Differences in the stamping frequencies of *daqu* lead to variations in the relationship between microorganisms and volatile compounds in *daqu*. From the perspective of bacteria, in YZ-5, *Lactobacillus*, *Bacillus*, and *Weissella* show a positive correlation with the vast majority of flavor compounds, including important aroma-producing compounds such as methyl hexanoate and methyl octanoate. As the stamping frequency decreases, the positive correlation of *Lactobacillus*, *Bacillus*, and *Weissella* with flavor compounds gradually weakens. In YZ-4, the positive correlation of *Lactobacillus* and *Bacillus* with flavor compounds weakens, and in YZ-3 and YZ-2, they show a negative correlation with flavor compounds. Previous studies have indicated that *Lactobacillus*, *Bacillus*, and *Weissella* are the main bacteria that produce flavor compounds in *daqu* [46]. Due to their anaerobic characteristics, they have a better growth rate in YZ-5 *daqu* with a smaller porosity, thus producing more ester compounds. As the stamping frequency decreases and the ester compounds in *daqu* decrease accordingly, we speculate that with the increase in porosity and the rise in oxygen content, the growth of lactic acid bacteria and *Bacillus* begins to be inhibited, and their aroma-producing ability also starts to decline.

From the aspect of fungi, *Saccharomycopsis*, *Candida*, and *Rhizomucor* in YZ-5 have a relatively weak correlation with flavor. However, as the stamping frequency decreases, the influence of these fungi on flavor gradually turns into a positive correlation. In YZ-2, the positive influence of *Saccharomycopsis*, *Candida*, and *Rhizomucor* on flavor increases, especially that *Candida* shows a positive correlation with the production of most ester compounds. To sum up, we speculate that as stamping frequency decreases, the dominant producer of flavor compounds in *daqu* shift from bacteria (lactic acid bacteria, *Bacillus*) and to fungi (yeasts, mucor). This shift may be caused by the difference in the oxygen content inside *daqu*, the fewer stamping frequencies, the more conducive it is for aerobic microorganisms to grow, the demand for oxygen by filamentous fungi is higher than that of lactic acid bacteria and *Bacillus*.

### 3.5. Prediction of Functional Enzymes in Daqu Across Stamping Frequencies

*Daqu* offers a variety of enzymes including those involved in carbohydrate hydrolysis, ethanol fermentation, and flavor formation [2]. These enzymes are likely to contribute to flavor formation during *baijiu* fermentation. Therefore, in this study, to explore the differences in enzyme functions of *daqu* with various stamping frequencies, 53 enzymes associated with carbohydrate hydrolysis, ethanol fermentation, and flavor formation in *daqu* were selected from the KEGG database and classified into 11 functional assemblies. The differences in the abundances of functional enzymes in *daqu* with different stamping frequencies were elucidated.

**Figure 7:**
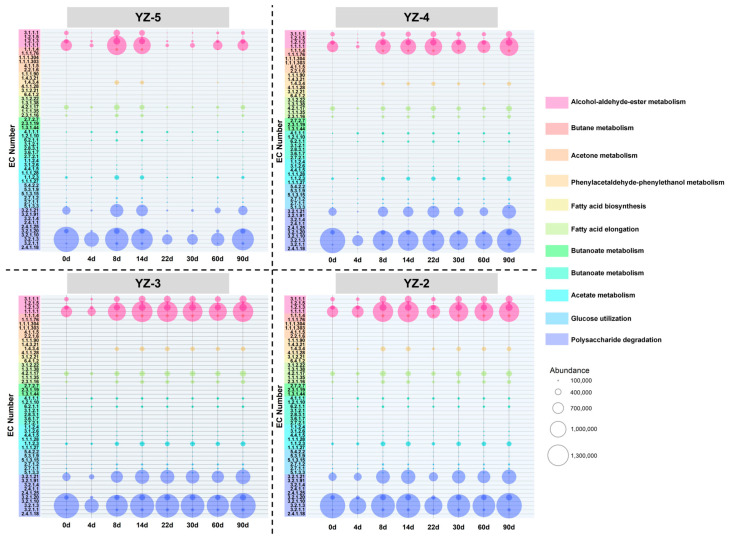
Abundance and functional classification of main enzymes in different stamping configurations *daqu*.

As depicted in Figure 7, the enzymatic functions of daqu with distinct stamping frequencies predominantly pertain to ethanol metabolism and polysaccharide metabolism. Specifically, alcohol dehydrogenase (EC: 1.1.1.1), aldehyde dehydrogenase (NAD(+)) (EC: 1.2.1.3), beta-glucosidase (EC: 3.2.1.21), and glucan 1,4-alpha-glucosidase (EC: 3.2.1.3) emerge as the preponderant functional enzymes within daqu. Nevertheless, the disparity in stamping frequency gives rise to variances in the abundance of these functional enzymes. In the case of YZ-5 daqu, the abundance of its functional enzymes is observed to be higher during the fermentation phase compared to the storage period. In contrast, the abundances of functional enzymes in the remaining daqu samples remain relatively stable. Concurrently, as the stamping frequency diminishes, the abundance of functional enzymes in daqu exhibits a progressive increment. Both alcohol dehydrogenase (EC: 1.1.1.1) and glucan 1,4-alpha-glucosidase (EC: 3.2.1.3) manifest a tendency wherein a lower stamping frequency corresponds to a greater abundance.

The abundance of enzymes is contingent upon the activity of microorganisms. Prior research has demonstrated that the enzymes associated with starch hydrolysis predominantly originate from *Aspergillus*, *Thermomyces* and *Thermoascus* [47,48]. As confirmed in Figure 3, YZ-5 contains a higher abundance of *Aspergillus* and *Thermoascus*, whereas YZ-4, YZ-3, and YZ-2 have a greater abundance of *Thermomyces*. Consequently, there were no significant differences in saccharifying power or the abundance of saccharifying enzymes among the four types *daqu* (Figure 7). Furthermore, the relatively lower abundance of alcohol dehydrogenase (EC: 1.1.1.1) in YZ-5 also renders ethanol less prone to decomposition within YZ-5, thereby causing the abundances of *Lactobacillus* and *Weissella* in YZ-5 to exceed those in the other groups (Figure 3). Previous studies have established that lactic acid bacteria constitute the most dominant microorganisms in the later stage of liquor fermentation and possess remarkable ethanol tolerance [49].

In summary, the stamping frequency exerts an influence on the abundance of microorganisms, thereby modifying the abundance of functional enzymes in *daqu*. Conversely, the abundance of functional enzymes also reciprocally impacts the microorganisms, culminating in disparate abundances of microorganisms among different *daqu* samples.

## 4. Conclusions

This study comprehensively analyzes *daqu* made by various stamping frequencies, including *daqu* physicochemical and biochemical indices, volatile compound profiles, microbial community dynamics, and functional enzyme activities, systematically evaluated the effects of mechanical stamping frequency on *daqu*.

At the macroscopic level, fewer stamping frequencies significantly reduced the retention of volatile compounds, leading to a gradual decrease in the concentration of ester substances. During the early fermentation stage of *nongxiangxing daqu* in multi-layered rack systems, the accumulation of metabolic heat accelerates moisture loss. In this case, increasing stamping frequency can alleviate this effect by reducing internal porosity, thereby slowing down water evaporation and maintaining stable physicochemical and biochemical indices.

At the microscopic level, stamping frequency significantly affected the microbial community composition and its functional associations with biochemical indices. Higher stamping frequencies selectively enriched lactic acid bacteria (*Weissella*, *Lactobacillus*) and filamentous fungi (*Aspergillus*), while fewer stamping frequencies promoted *Rhizopus* and yeast species to become dominant groups. The above results show that with the decrease in stamping frequency, the dominant of physicochemical and flavor substances in daqu is changed from bacteria to fungi.

The above findings provide theoretical guidance for *daqu* production: producers can regulate physicochemical and biochemical indices as well as microbial composition by adjusting stamping frequency in combination with the process mode, thereby optimizing the flavor quality of the final *baijiu*.

To further extend the practical value of these findings, subsequent research could explore scaling up fermentation processes to bridge laboratory observations with industrial production contexts. Additionally, integrating metabolomics approaches may help substantiate the functional implications of microbial community shifts, while sensory evaluation studies could better connect daqu properties with consumer perceptions of the final baijiu, collectively refining the application of stamping frequency regulation in production practice.

## Data Availability

Data presented in this study are available upon request from the corresponding authors upon reasonable request.

## Supplementary Materials

The following supporting information can be downloaded at https://www.mdpi.com/article/10.3390/foods14213700/s1, Figure S1: Schematic diagram of making Daqu with different stamping configurations; Figure S2: Initial density of Daqu with different stamping frequency; Figure S3: α-diversity of Daqu with different stamping frequencies; Table S1: Volatile compounds in different stamping frequencies daqu.

## Abbreviations

The following abbreviations are used in this manuscript:

HS-SPME-GC-MS	Headspace solid-phase microextraction gas chromatography–mass spectrometry
