# Supplementary material for: Mining the Impact of Mechanical-Stamping Heterogeneity on the Macro- and Micro-Levels of Nongxiangxing daqu
Source: Foods. 2025 Oct 29;14(21):3700. doi: 10.3390/foods14213700 (PMC12607447; doi:10.3390/foods14213700)
Supplement: Supplementary file 1 [file foods-14-03700-s001.zip › Supplementary.pdf]

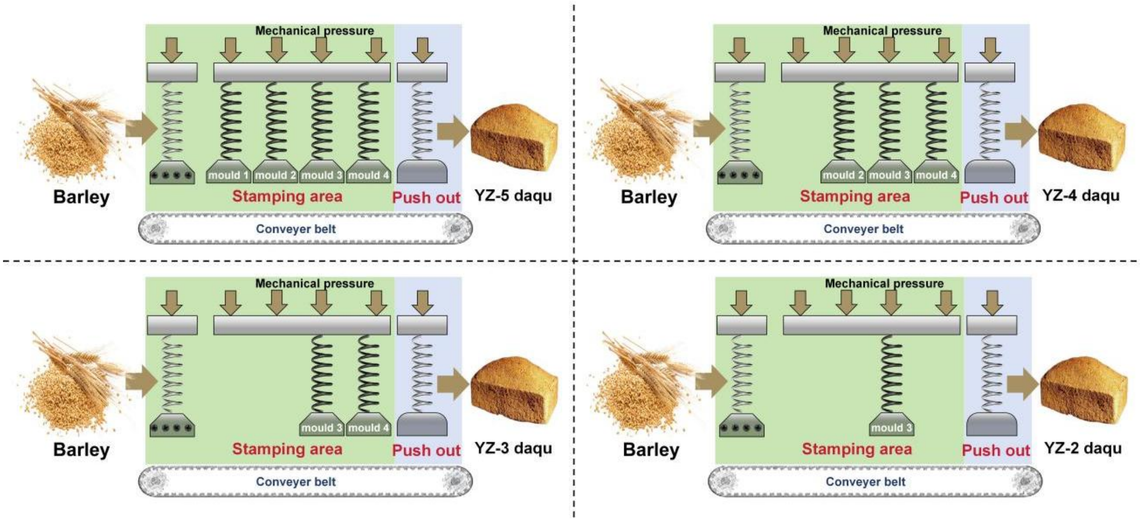

Figure S1 Schematic diagram of making Daqu with different stamping configurations

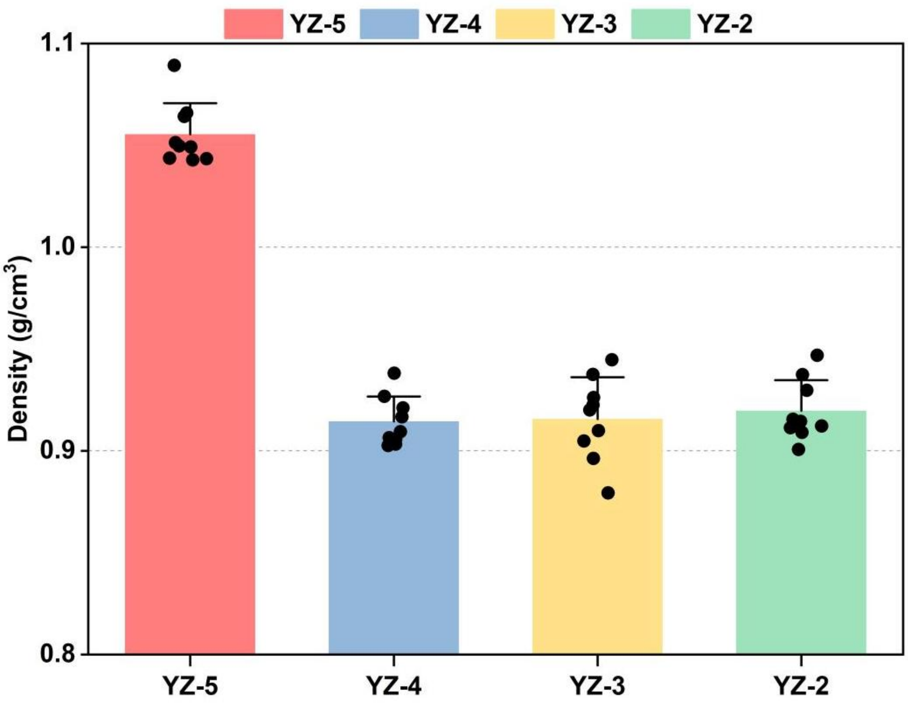

Figure S2 Initial density of Daqu with different pressing times

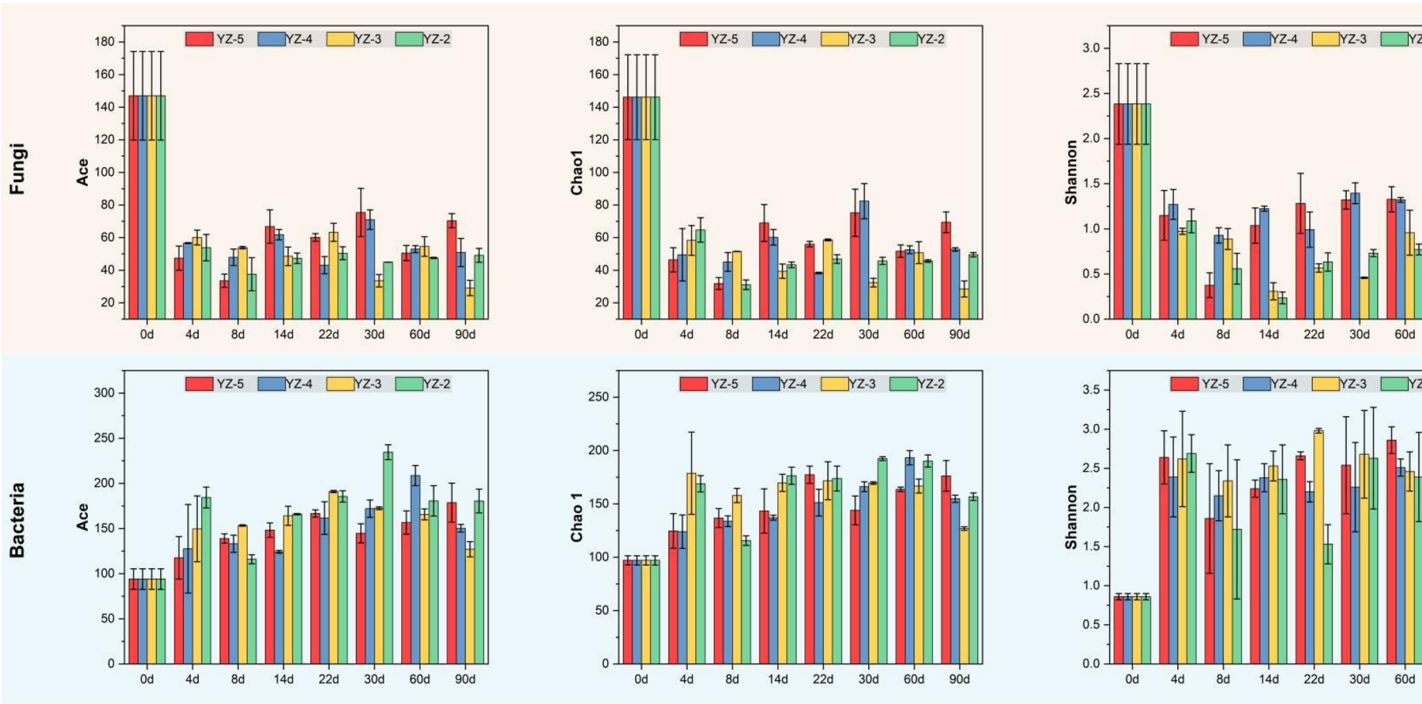

Figure S3 α-diversity of Daqu with different stamping frequencies
